# PW02-028 - Association of novel NLRP3 mutations with CAPS phenotype in Turkish patients

**DOI:** 10.1186/1546-0096-11-S1-A169

**Published:** 2013-11-08

**Authors:** A Berdeli, S Nalbantoglu, D Tigli, I Demirel, M Atan, B Sozeri

**Affiliations:** 1 Molecular Medicine and Pediatric Rheumatology, Izmir, Turkey; 2 Molecular Medicine Laboratory, Izmir, Turkey; 3 Pediatric Rheumatology, EGE UNIVERSITY, Izmir, Turkey

## Introduction

Cryopyrin-Associated Periodic Syndromes (CAPS) are a group of rare, inherited, autoinflammatory diseases involved of Familial Cold Autoinflammatory Syndrome (FCAS),Muckle-Wells Syndrome (MWS) and Neonatal Onset Multisystem Inflammatory Disease (NOMID) (also called Chronic Infantile Neurologic Cutaneous Articular, or CINCA, Syndrome. The responsible gene NLRP3 (nucleotide-binding domain, leucine-rich family [NLR], pyrin domain containing, produces cryopyrin protein which participates in inflammasome complexes leading to production of interleukin-1β (IL-1β) and autoinflammation.

## Objectives

We aimed to investigate possible variations of the genes linked to CAPS disorders.

## Methods

Bidirectional DNA Sequencing analysis was performed in coding exons and exon-intron flanking regions of NLRP3 gene (NM_004895.4; NP_004886.3).

## Results

Patients with manifestations of hereditary autoinflammatory disorder and MEFV gene mutation negative (n:194) had undergone mutation analysis of NLRP3 gene. Disease related mutations were obtained in 25 patients. p.Gln703Lys in 19 patients, p.Ser726Gly in 2 patients and p.Val198Met mutation in 1 patient were investigated. In 3 patients, novel pathogenic p.Ala154Gly, p. Ser726Gly missense, and p.K610fsX613 frameshift mutations were identified, and registered to ISSAID. Synonimous amino acid polymorphisms include: p.Thr219Thr (ACC/ACT; c.657C>T) %14, p.Ala242Ala (GCG/GCA; C.726G>A) %56.4, p.Arg260Arg (CGA/CGG; c.780A>G) %63.6, p.Ser434Ser (TCC/TCT; c.1302C>T) %23.4 and p.Leu411Leu (c.1231C-T) %0.4. Anti-IL1B treatment was started for those patients and good response was obtained.

## Conclusion

In this study, DNA sequencing analysis revealed novel NLPR3 mutations which were found related to typical CAPS phenotype and registered to ISSAID. In particular, for patients with no identifiable MEFV gene mutation, genetic diagnosis helps early diagnosis, proper follow-up and treatment of patients in CAPS disorders. For the genetic and phenotypic heterogeneity of the patients and for patients with no identifiable mutations, possible presence of other genetic and non-genetic factors should be investigated.

## Disclosure of interest

None declared.

